# Dulaglutide Modulates the Development of Tissue-Infiltrating Th1/Th17 Cells and the Pathogenicity of Encephalitogenic Th1 Cells in the Central Nervous System

**DOI:** 10.3390/ijms20071584

**Published:** 2019-03-29

**Authors:** Hsin-Ying Clair Chiou, Ming-Wei Lin, Pi-Jung Hsiao, Chun-Lin Chen, Shiang Chiao, Ting-Yi Lin, Yi-Chen Chen, Deng-Chyang Wu, Ming-Hong Lin

**Affiliations:** 1 Division of Endocrinology and Metabolism, Department of Internal Medicine, Kaohsiung Medical University Hospital, Kaohsiung City 807, Taiwan; phoenixchiou@gmail.com (H.-Y.C.C.); pjhsiao101@gmail.com (P.-J.H.); 2 Department of Medical Research, E-Da Hospital/E-Da Cancer Hospital, Kaohsiung City 824, Taiwan; ta990074@gmail.com; 3 School of Medicine, College of Medicine, I-Shou University, Kaohsiung City 824, Taiwan; 4 Center for Stem Cell Research, Kaohsiung Medical University, Kaohsiung City 807, Taiwan; dechwu@yahoo.com; 5 Department of Internal Medicine, School of Medicine, College of Medicine, Kaohsiung Medical University, Kaohsiung City 807, Taiwan; 6 Department of Biological Science, National Sun Yat-Sen University, Kaohsiung City 804, Taiwan; chunlinchen@mail.nsysu.edu.tw; 7 Department of Microbiology and Immunology, School of Medicine, College of Medicine, Kaohsiung Medical University, Kaohsiung City 807, Taiwan; jjiimm0468@gmail.com (S.C.); lintingyi2014@gmail.com (T.-Y.L.); joanna830615@yahoo.com.tw (Y.-C.C.); 8 Graduate Institute of Medicine, College of Medicine, Kaohsiung Medical University, Kaohsiung City 807, Taiwan; 9 Division of Gastroenterology, Department of Internal Medicine, Kaohsiung Medical University Hospital, Kaohsiung City 807, Taiwan; 10 M.Sc. Program in Tropical Medicine, College of Medicine, Kaohsiung Medical University, Kaohsiung City 807, Taiwan; 11 Department of Medical Research, Kaohsiung Medical University Hospital, Kaohsiung City 807, Taiwan

**Keywords:** Experimental autoimmune encephalomyelitis, dulaglutide, GLP-1 RA, autoreactive T cells, Th1, Th17, encephalitogenicity, dendritic cells (DCs), macrophages, multiple sclerosis, central nervous system

## Abstract

GLP-1 (glucagon-like peptide-1) has been reported to play a vital role in neuroprotection. Experimental autoimmune encephalomyelitis (EAE) is a well-established animal model widely used to study human multiple sclerosis, a chronic demyelination disease in the central nervous system (CNS). Recently, important studies have designated that the signaling axis of GLP-1 and its receptor controls the clinical manifestations and pathogenesis of EAE. However, it is elusive whether GLP-1 receptor signaling regulates the phenotype of autoreactive T cells in the CNS. We administered dulaglutide, a well-established GLP-1 receptor agonist (GLP-1 RA), to treat EAE mice prophylactically or semi-therapeutically and subsequently analyzed the mononuclear cells of the CNS. In this study, dulaglutide treatment significantly alleviates the clinical manifestations and histopathological outcomes of EAE. Dulaglutide decreases incidences of encephalitogenic Th1/Th17 cells and Th1 granulocyte-macrophage-colony-stimulating factor (GM-CSF) expression in the CNS. Administration of dulaglutide failed to control the chemotactic abilities of encephalitogenic Th1 and Th17 cells; however, prophylactic treatment considerably decreased the populations of dendritic cells and macrophages in the CNS parenchyma. These results obtained indicate that dulaglutide modulates the differentiation of encephalitogenic Th1/Th17 and the pathogenicity of Th1 cells by influencing antigen presenting cells quantities, providing mechanism insight on T cells regulation in ameliorating EAE by GLP-1.

## 1. Introduction

GLP-1 (glucagon-like peptide-1) is best known as the hormone that induces insulin release during hyperglycemia. Vital for neuroprotection [1], the signaling axis of GLP-1 and its receptor, GLP-1R, is involved in the pathogenesis of several central nervous system (CNS) disorders [1]. Accumulating data revealed that the administration of GLP-1 receptor agonist (GLP-1 RA) not only alleviates traumatic or ischemic brain damage [2,3] but also ameliorates neurodegenerative diseases demonstrated in Alzheimer’s disease (AD) and Parkinson’s disease mice models [4,5] through reduced neuronal cell death and microglial activation. Since the GLP-1 receptor is expressed throughout the brain, GLP-1 RA is considered to have neurotrophic and anti-inflammation effects for CNS diseases [2,3,4,5,6]. Quite recently, it has been proved that the administration of GLP-1 RA delays the disease onset of experimental autoimmune encephalomyelitis (EAE) in Lewis rats by dampening the effects of oxidative stress [7]. Consistently, activation of GLP-1 receptor signaling attenuates the clinical severity and incidence of EAE, partially via the inactivation of NF-κB signaling in spinal cord and microglia cells [8]. However, whether GLP-1 RA modulates autoreactive T cell subsets and their pathogenicity during neuro-inflammation of EAE remains to be elucidated.

Multiple sclerosis (MS) is a chronic demyelinating autoimmune disease of the CNS caused by autoreactive T cells, and EAE is a well-established mouse model for the investigation of human MS [9]. In the pathogenesis of EAE [10], both Type 1 helper T (Th1) and Type 17 helper T (Th17) cells, also known as interferon (IFN)-γ-secreting and interleukin (IL)-17A-producing CD4-positive T cells respectively, are critical inflammatory T cells that mediate the demyelination of neurons in the CNS and consequently, mediate the ascending paralysis of tail and limbs of mouse [11,12,13]. CNS-infiltrating IFN-γ/IL-17A-doubly-secreting CD4 T (Th1/Th17) cells are indispensable players in the pathogenesis of EAE [14]. Furthermore, several studies confirm that the granulocyte-macrophage-colony-stimulating factor (GM-CSF) and tumor necrosis factor (TNF)-α secreted by Th1 or Th17 cells also induce autoimmune encephalomyelitis [15,16,17]. Despite substantial evidence reinforcing Th1/Th17 cells as pro-EAE, expanding knowledge of T cell subsets properties challenge the hypothesis mentioned above. IL-10 productions in Th1 [18] or Th17 [19] effector cell subset commit a T regulatory type 1 (Tr1)-like phenotype and contribute to the resolution of inflammation in the CNS. Chemokine receptors, CXCR3 and CCR6, are critical regulators for the migration of encephalitogenic Th1 [20] and Th17 [21] cells, respectively, into inflammatory lesions of CNS in autoimmune encephalomyelitis. Macrophages [22] and dendritic cells (DCs) [23] are involved in the processes of priming CD4-positive T cell subsets during inflammation responses in the CNS of diseases.

GLP-1 signaling is implicated in lymphocyte proliferation and regulatory T cell maintenance [24]. Upon mitogen stimulation, the *Glp1r-/-* thymocytes show hypoproliferation whilst the peripheral *Glp1r-/-* lymphocytes were hyperproliferative. Moreover, low percentage of the regulatory T cells were found in male *Glp1r-/-* mice, although the CD4+ and CD8+ T cells and B cells were not altered in the spleen and lymph nodes [24]. Treatment of GLP-1 RA on NOD mice, which exhibits spontaneous type 1 diabetes, increases the frequency of regulatory T cells [25]. In addition, activation of the GLP-1 receptor on the intestinal intraepithelial lymphocyte suppresses the pro-inflammatory cytokine expression [26]. Here, by using EAE mice model, which is a T cell- driven autoimmune disease, we aim to test whether GLP-1 RA regulates autoreactive T cell subsets and their development as well as pathogenicity in the CNS., The cytokine expressions and chemotactic abilities of each T cell subset were analyzed. Moreover, the dendritic cells and macrophage, which is responsible for T cell activation were also analyzed in this study.

## 2. Results

### 2.1. Dulaglutide Treatment Significantly Attenuates the Clinical Manifestations and Histopathological Outcomes of EAE

**Figure 1:**
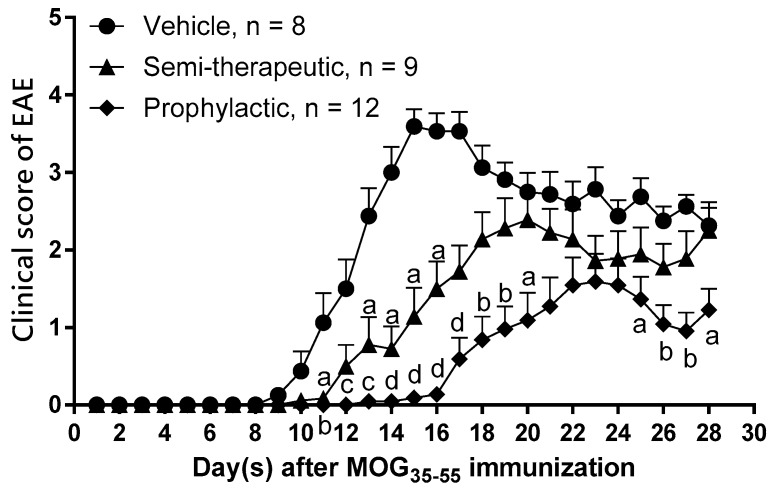
GLP-1 receptor agonist (GLP-1 RA) dulaglutide treatment significantly attenuates the pathogenic processes of myelin oligodendrocyte glycoprotein (MOG)_35–55_-induced experimental autoimmune encephalomyelitis (EAE). Clinical scores of EAE in mice treated with prophylactic (filled diamond) or semi-therapeutic (filled triangle) dulaglutide administration. EAE mice received treatment of saline served as vehicle control (filled circle) (*n* = 8). In the group prophylactic treatment, EAE mice were administrated with dulaglutide at 0, 3, 7, and 10 days after MOG_35–55_ immunization (*n* = 9). In the group of semi-therapeutic treatment, EAE mice were treated with dulaglutide at 9, 12, 16, 19, 23, and 26 days after MOG_35–55_ immunization (*n* = 12). All data are representative of three independent experiments and were presented as mean ± SEM from, at least, eight mice in each group. Alphabet a, *p* < 0.05; b, *p* < 0.01; c, *p* < 0.001; d, and *p* < 0.0001 was analyzed by nonparametric Kruskal–Wallis test followed by post-hoc test, Dunn’s multiple comparisons test.

The signaling axis of GLP-1 and its receptor is critical in the pathogenesis of EAE [6,7]. To test the immune modulation of dulaglutide, a GLP-1 RA, in autoimmune encephalomyelitis, we immunized C57BL/6 mice with MOG_35–55_/CFA (complete Freund’s adjuvant) emulsion and pertussis toxin to induce EAE. Respectively, these MOG-immunized mice were administered with saline, prophylactic, or semi-therapeutic treatment by dulaglutide. Our current results indicated that the clinical score of EAE was significantly attenuated in mice treated with prophylactic or semi-therapeutic dulaglutide as compared to vehicle mice, respectively (Figure 1). The disease onset day of EAE was significantly delayed in mice treated with prophylactic (18.82 ± 1.256) or semi-therapeutic (14.00 ± 0.7601) dulaglutide as compared to vehicle mice (10.88 ± 0.5154), respectively (Table 1). Concurring with the protective role of dulaglutide, maximal clinical scores of EAE were likewise diminished in prophylactic (2.021 ± 0.3053) or semi-therapeutic (2.889 ± 0.2170) group as compared to the vehicle group (3.781 ± 0.2083), respectively (Table 1). Although the clinical severity and disease onset shifted favorably, the disease interval where EAE mice suffer from maximal clinical score remained unswerving among vehicle group (2.375 ± 0.3239), prophylactic (2.7 ± 0.5175) and semi-therapeutic (2.0 ± 0.2887) dulaglutide treatment (Table 1).

**Figure 2:**
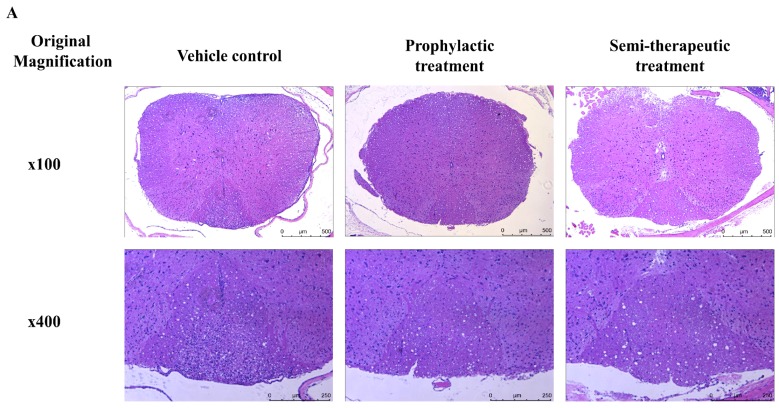

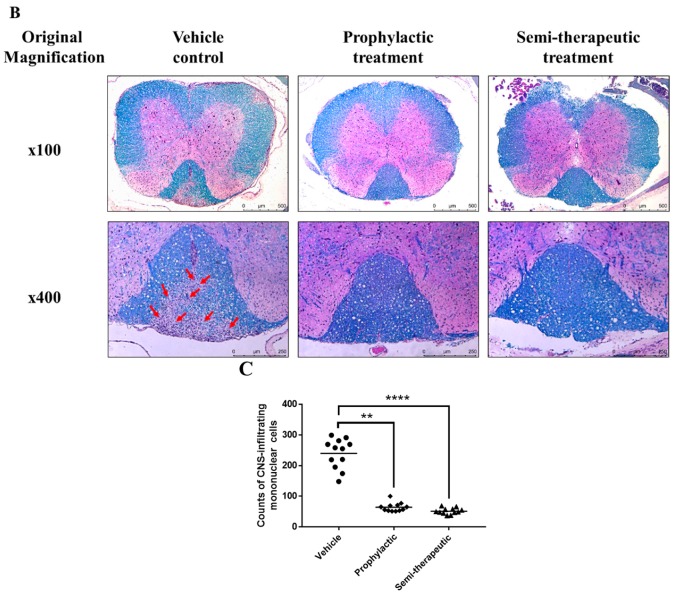
GLP-1 RA dulaglutide treatment obviously alleviates the lymphocyte infiltration, vacuolar degeneration, and neuronal demyelination in the CNS of MOG-immunized mice. (**A**) Analysis of hematoxylin and eosin (H&E) stain of the spinal cord tissue section. (**B**) Analysis of Luxol Fast Blue (LFB) stain of the spinal cord tissue section. Spinal cords were removed from mice at day 14 after MOG_35–55_ immunization. Spinal cords were analyzed with hematoxylin and eosin stain. Demyelinated areas were indicated as red arrows. (**A**,**B**) Representative results of prophylactic, semi-therapeutic dulaglutide treatment and vehicle control were showed. Upper row was showed as magnification x100 and the lower row was showed as x400. Each group had three mice for H&E stain and LFB stain, respectively. (**C**) Cell counts of central nervous system (CNS)-infiltrating mononuclear cells from the dorsal white matter of spinal cord in EAE mice received vehicle (filled circle), prophylactic (filled diamond), or semi-therapeutic (filled triangle) treatment of dulaglutide. Four non-redundant regions with 400x high power magnification were acquired from individual mouse and then further calculated by Image J software. All data are representative of three mice in each group and presented as mean. ** *p* < 0.01 and **** *p* < 0.0001 by the non-parametric Kruskal–Wallis test followed by post-hoc test, Dunn’s multiple comparisons test.

To further confirm histological results of tissue sections, we used cervical spinal cord collected from MOG-immunized mice treated with prophylactic, semi-therapeutic dulaglutide treatment or vehicle control at day 14 after MOG immunization. Hematoxylin and eosin (H&E) stain results revealed that lymphocyte infiltrations and vacuolar degenerations of dorsal as well as ventral funiculus were alleviated in EAE mice treated with either prophylactic or semi-therapeutic dulaglutide administration as compared to vehicle control, respectively (Figure 2A,C). Furthermore, we used Luxol Fast Blue (LFB) stain combined with H&E stain to detect demyelination in the CNS of EAE mice. Demyelination of cervical spinal cord, especially in dorsal funiculus, was rescued in MOG-immunized mice administrated with either prophylactic or semi-therapeutic dulaglutide treatment as compared to vehicle control, respectively (Figure 2B). These histopathological results were consistent with the disease progression of EAE in mice identified previously and establishes the value of dulaglutide as a robust immune-modulatory target for tackling autoimmune encephalomyelitis.

### 2.2. Dulaglutide Administration Markedly Downregulates the Development of Encephalitogenic Th1/Th17 Cell Subsets in the CNS of MOG-Immunized Mice

**Figure 3:**
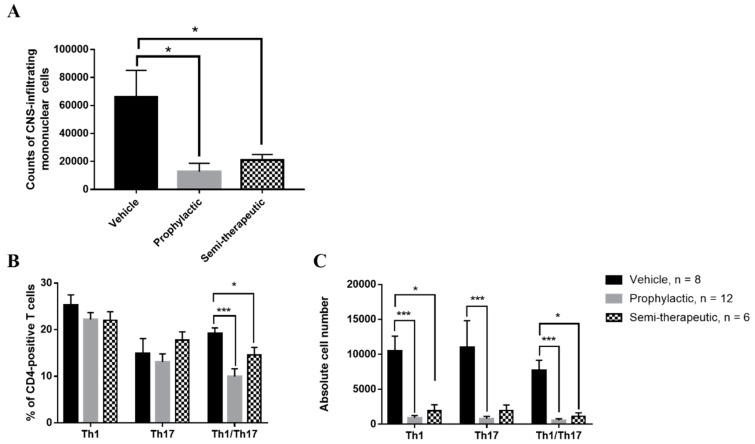
GLP-1 RA dulaglutide administration notably inhibits the development of tissue-infiltrating interferon (IFN)-γ/interleukin (IL)-17A doubly-secreting encephalitogenic CD4-positive T cell subset in the CNS. (**A**) Absolute cell numbers of CNS-infiltrating CD45^hi^ mononuclear cells in EAE mice. Percentages (**B**) and absolute numbers (**C**) of Th1, Th17 cells, and IFN-γ/IL-17A double-producing CD4+ T cells (Th1/Th17) in CNS of EAE mice, respectively. EAE mice, treated with prophylactic (gray bar), semi-therapeutic (dotted bar) dulaglutide, or vehicle control (black bar), were analyzed at day 14 after MOG immunization. Mononuclear cells were isolated from CNS of EAE mice and subjected to the analysis of intracellular cytokine staining and flow cytometry. The data are representative of three independent experiments. All data for percentage and absolute number were presented as mean ± SEM from, at least, 6 mice in each group. **** *p* < 0.0001; *** *p* < 0.001; ** *p* < 0.01; * *p* < 0.05 by non-parametric Kruskal–Wallis test followed by post-hoc analysis, Dunn’s multiple comparisons test.

Several authors have vigorously proposed that the encephalitogenic Th1 and Th17 cells are to be blamed for the pathogenesis of EAE [9]. The most well-known advocating evidence of this statement is the development of Th1/Th17 CNS-infiltrating cells in the CNS and the causal exacerbation of EAE progression due to their presence [13]. In an attempt to examine the influence of dulaglutide on the development of proinflammatory T cell subsets in CNS, we isolated CNS-infiltrating mononuclear cells from the pooled brain and spinal cord samples and analyzed the percentages and absolute cell numbers of CD4-positive T cell subsets. We found that the absolute cell numbers of CD45^hi^–expressing CNS-infiltrating mononuclear cells were significantly lower in mice treated with prophylactic and semi-therapeutic dulaglutide administration than those mice treated with vehicle control (Figure 3A), respectively. Our data revealed that both percentages of Th1 and Th17 cells are statistically insignificant in the CNS of EAE mice that received the vehicle, prophylactic, or semi-therapeutic treatment, respectively. Nevertheless, the frequency of IFN-γ/IL-17A doubly-producing CD4+ T cell (Th1/Th17) subset was significantly declined in the CNS of MOG-immunized mice treated with prophylactic or semi-therapeutic dulaglutide administration as compared to vehicle control (Figure 3B). Mean fluorescence index (MFI) of cytokines in those T helper cells had no significant variation. The exploration that dulaglutide explicitly affects the development of Th1/Th17 cell subset without impairing the quantities of cytokine production in cells and no single populations brings to light the role of GLP1 in mitigating doubly Th1/Th17 immunomodulation. Moreover, we found that absolute cell numbers of Th1 and Th1/Th17 cells were drastically decreased in the CNS of EAE mice that received the administration of prophylactic or semi-therapeutic dulaglutide as compared to vehicle control, respectively (Figure 3C). Nevertheless, the cell number of encephalitogenic Th17 cells was only diminished when treated with prophylactic dulaglutide, but not semi-therapeutic group as compared to vehicle control, respectively (Figure 3C), highlights the importance of timing in GLP1 immunomodulation. Taken together, our results suggested that dulaglutide administration may regulate the differentiation of tissue-infiltrating Th1/Th17 cell subset and affect the numbers of encephalitogenic T cell subsets in the CNS of EAE mice.

### 2.3. Prophylactic Dulaglutide Treatment Potently Diminishes the GM-CSF Production of Encephalitogenic Th1 Cells in CNS

**Figure 4:**
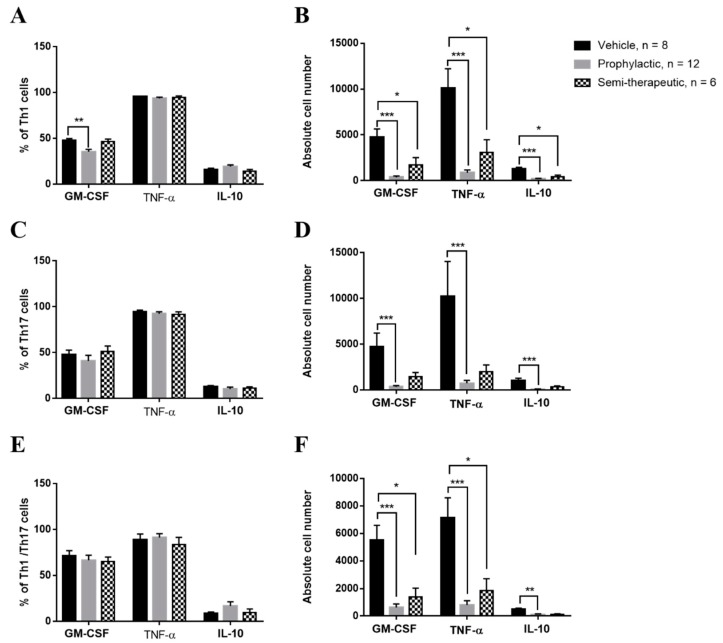
Prophylactic GLP-1 RA dulaglutide treatment strikingly diminishes the frequency of granulocyte-macrophage-colony-stimulating factor (GM-CSF)-producing encephalitogenic Th1 cell subset in the CNS of MOG-immunized mice. Frequency (**A**,**C**,**E**) and cell number (**B**,**D**,**F**) of GM-CSF-, Tumor necrosis factor α (TNF-α)-, or IL-10-producing in Th1 (**A**,**B**), Th17 (**C**,**D**), or Th1/Th17 (**E**,**F**) cells from CNS of EAE mice, respectively. MOG-immunized mice were treated with indicated regimens, including prophylactic (gray bar), semi-therapeutic (dotted bar) treatment, or vehicle control (black bar) treatment. At day 14 after MOG immunization, mononuclear cells were isolated from CNS of EAE mice and were analyzed by the procedure of intracellular cytokine staining. The data are representative of three independent experiments. All data for frequency and cell number were presented as mean ± SEM from, at least, 6 mice in each group. *** *p* < 0.001; ** *p* < 0.01; * *p* < 0.05 by the non-parametric Kruskal–Wallis test followed by post-hoc analysis, Dunn’s multiple comparisons test.

Proven unambiguously, GM-CSF production from encephalitogenic Th1 and Th17 cell subsets are paramount in accelerating pathogenicity via neuroinflammation of the CNS [14,15,16]. To investigate whether dulaglutide treatment modulated the encephalitogenicity of T cell subsets via the control of GM-CSF, TNF-α and IL-10, we analyzed these cytokines in encephalitogenic Th1, Th17, and Th1/Th17 cell subsets in the CNS of MOG-immunized mice, respectively. Mice treated with dulaglutide presented with the significantly diminished percentage of GM-CSF-producing Th1 cell subset (Figure 4A), revealing a suppressive role of dulaglutide in the development of GM-CSF-positive Th1 cells. However, the frequency of GM-CSF-secreting Th1 cells remains constant in EAE mice treated with semi-therapeutic dulaglutide administration as compared to vehicle treatment, despite an increase in MOG-immunized mice administrated with semi-therapeutic dulaglutide as compared to prophylactic treatment (Figure 4A). Briefly, these results indicated that only prophylactic dulaglutide treatment may alleviate the encephalitogenicity of Th1 cell subset in the CNS of EAE mice via downregulation of GM-CSF. We also measured the frequency of TNF-α or IL-10 in CNS-infiltrating Th1 cell subset among three treatments as mentioned above. These results indicated that both the percentages of TNF-α and IL-10 in encephalitogenic Th1 cells were similar among EAE mice receiving the three distinct treatments, respectively (Figure 4A). We further found that absolute cell number of GM-CSF, TNF-α, or IL-10-secreting Th1 cells was dramatically diminished in the CNS of EAE mice received either prophylactic or semi-therapeutic dulaglutide treatment as compared to vehicle control, respectively (Figure 4B). These results suggested that dulaglutide treatment potently attenuated the encephalitogenicity of Th1 via downregulation of GM-CSF production. Dulaglutide administering appreciably reduced the absolute numbers of proinflammatory GM-CSF or TNF-α-producing Th1 cells in the CNS of EAE mice; moreover, the number of tolerogenic IL-10-secreting Th1 cells were also decreased. For encephalitogenic Th17 and Th1/Th17 cell subsets, we found that the percentage of GM-CSF, TNF-α or IL-10-producing Th17 or Th1/Th17 cells were similar in the CNS of MOG-immunized mice received vehicle control, prophylactic, or semi-therapeutic dulaglutide treatment, respectively (Figure 4C,E). Analysis of the cell numbers of GM-CSF, TNF-α, or IL-10-secreting Th17 or Th1/Th17 cell subset in the CNS. Our results revealed that cell numbers of those Th17 cell subsets were significantly decreased in the CNS of EAE mice with prophylactic dulaglutide treatment as compared to vehicle control, respectively (Figure 4D). Nonetheless, the analysis did not identify any significant differences between the semi-therapeutic dulaglutide and vehicle treatment groups. (Figure 4D). For encephalitogenic Th1/Th17 cell subset, our results revealed that absolute number of GM-CSF, TNF-α, or IL-10-producing cells was markedly reduced in the CNS of EAE mice treated with either prophylactic or semi-therapeutic dulaglutide administration as compared to vehicle control, respectively (Figure 4F). We re-confirmed that the quantities of cytokines, for example IFN-γ, IL-17A, GM-CSF, TNF-α, and IL-10, in those T helper cell subsets as mentioned above (Figure 4A,C,E) were similar among EAE mice received different regimens of dulaglutide. Conclusively, our results further suggested that prophylactic dulaglutide treatment may potently constrict the cell numbers of highly encephalitogenic Th1, Th17, and Th1/Th17 cell subsets in CNS of EAE mice, as well as semi-therapeutic treatment, may only affect the numbers of Th1 and Th1/Th17 cell subsets.

### 2.4. Dulaglutide Treatment Inefficiently Controls the Chemotaxis of Encephalitogenic Th1 and Th17 Cells into the CNS of MOG-Immunized Mice

**Figure 5:**
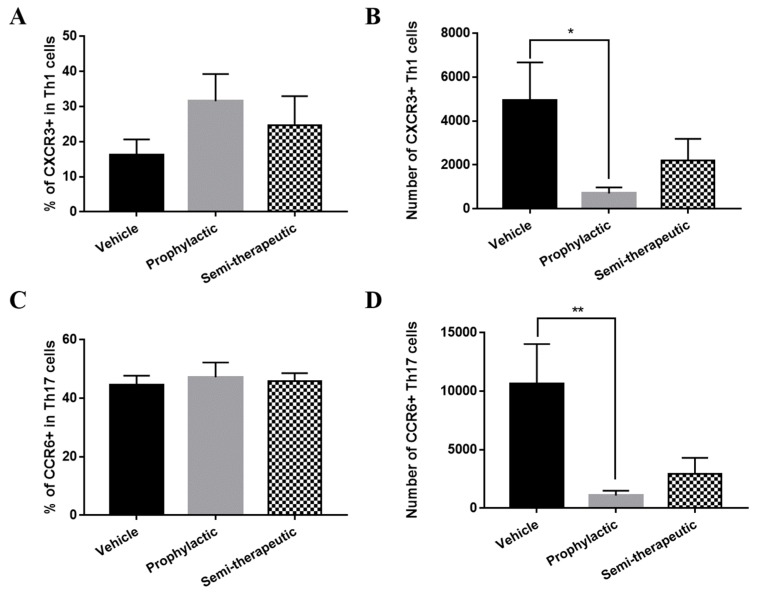
GLP-1 RA dulaglutide treatment inefficiently affects the chemotactic abilities of encephalitogenic Th1 as well as Th17 cells into the CNS. Frequency (**A**,**C**) and absolute number (**B**,**D**) of CXCR3+ Th1 (**A**,**B**) and CCR6+ Th17 (**C**,**D**) cells in CNS of EAE mice, respectively. EAE mice were treated with three different regimens, including prophylactic (gray bar), semi-therapeutic (dotted bar), and vehicle control (black bar) treatments. Mononuclear cells were isolated from CNS of MOG-immunized mice at day 14 after EAE induction. CXCR3 was represented as a chemo-attractive receptor for Th1 cells; nevertheless, CCR6 was representative of the chemo-attractant receptor for Th17 cells. Cells were verified by the analysis of flow cytometry. The data are representative of three independent experiments. All data for frequency and cell number were presented as mean ± SEM from, at least, 6 mice in each group. * *p* < 0.05 and ** *p* < 0.01 by the non-parametric Kruskal–Wallis test followed by post-hoc analysis, Dunn’s multiple comparisons test.

After establishing qualitatively and quantitatively the constitution and fidelity of T cell subset populations upon dulaglutide treatment, we wonder whether the effect of prophylaxis stems from T cell chemotaxis. Established by prior literatures, CXCR3 and CCR6 are crucial chemotactic factors for encephalitogenic Th1 and Th17 cell subset toward CNS [19,20]. To explore whether dulaglutide administration affected the chemotaxis of encephalitogenic Th1 and Th17 cells in the CNS lesion of EAE mice, we measured the frequencies of CXCR3 in Th1 cells and CCR6 in Th17 cells of the CNS from MOG-immunized mice with the treatment of vehicle control, prophylactic, or semi-therapeutic manner. Our results revealed nonsignificant alterations in the percentages of CXCR3 in encephalitogenic Th1 cells and CCR6 in Th17 cells (Figure 5A,C). In those Th1 and Th17 cells, the amounts of CXCR3 and CCR6 were the same from EAE mice treated with different regimens, respectively. These results shed light on the lack of involvement of GRP-1 signaling in the chemo-attractive migration of encephalitogenic T cell subsets and welcome an alternative mechanism. Nevertheless, when comparing the cell numbers of CXCR3-positive Th1 and CCR6-positive Th17 in the CNS of EAE mice upon the treatment of vehicle control, prophylactic, or semi-therapeutic dulaglutide; Figure 5B,D showed that prophylactic but not semi-therapeutic dulaglutide administration significantly decreased the absolute numbers of CXCR3-positive Th1 cells and CCR6-positive Th17 cells in the CNS of EAE mice, despite constant frequencies of these subsets. The exploration on the grounds of chemotaxis, overall, emphasized the absence of GRP-1 engagement by revealing that dulaglutide administration does not regulate the migration potential of encephalitogenic T cells into the CNS to elicit the pathogenic progression of autoimmune encephalomyelitis.

### 2.5. Prophylactic Dulaglutide Administration Strikingly Alleviates the Absolute Numbers of Dendritic Cells and Macrophages in the CNS of EAE Mice

**Figure 6:**
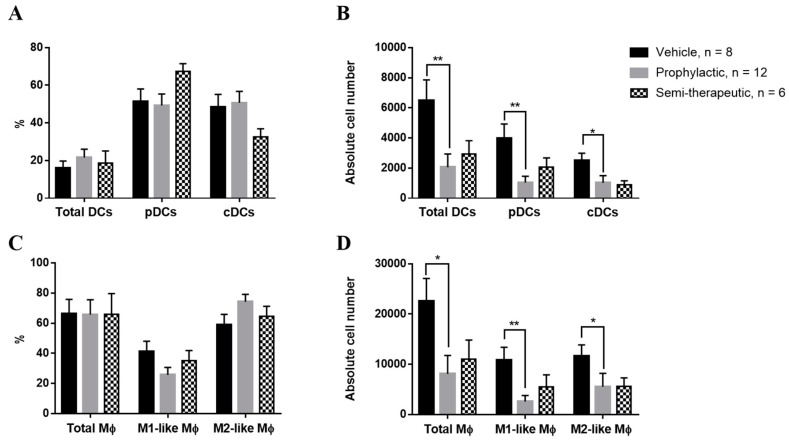
GLP-1 RA dulaglutide administration markedly decreases the numbers of dendritic cells (DCs) and macrophages in the CNS parenchyma of EAE mice. Percentages (**A**,**C**) and absolute numbers (**B**,**D**) of dendritic cell (DC) (**A**,**B**) and macrophage (Mψ) (**C**,**D**) populations in CNS of EAE mice, treated with prophylactic (gray bar), semi-therapeutic (dotted bar) treatment, or vehicle control (black bar) treatment. (**A**,**B**) Percentages and cell numbers of total dendritic cells (DCs: I-A/I-E+/F4/80-/CD11b+/CD11c+), plasmacytoid dendritic cells (pDCs: I-A/I-E+/F4/80-/CD11b+/CD11c+/B220+), or classic dendritic cells (cDCs: I-A/I-E+/F4/80-/CD11b+/CD11c+/B220-). (**C**,**D**) Percentages and cell numbers of total macrophages (I-A/I-E+/F4/80+/CD11b+), M1-like macrophages (I-A/I-E+/F4/80+/CD11b+/CD11c+), or M2-like macrophages (I-A/I-E+/F4/80+/CD11b+/CD11c-). EAE mice, treated as indicated regimens, were sacrificed at day 14 after MOG immunization. Mononuclear cells were purified from CNS of EAE mice and later CD45^hi^ cells were analyzed by the procedure of surface marker staining. The data were collected from three independent experiments. All data for frequency and cell number were presented as mean ± SEM from, at least, 6 mice in each group. ** *p* < 0.01; * *p* < 0.05 by non-parametric Kruskal–Wallis test followed by post-hoc test, Dunn’s multiple comparisons analysis.

DCs and macrophages are key antigen-presenting cells (APCs) during the pathogenesis of EAE [10,22,27]. To simulate the real-life situation of an orchestrated immune response, we measured the percentage and absolute numbers of DCs and macrophages in the CNS of EAE mice with the treatment of vehicle control, prophylactic, or semi-therapeutic dulaglutide administration. We found that dulaglutide treatments had no effect on the percentages of DCs subsets, as compared to vehicle control (Figure 6A). However, our results emphasized that the absolute numbers of total DCs (I-A/I-E+/F4/80-/CD11b+/CD11c+), plasmacytoid DCs (pDCs, I-A/I-E+/F4/80-/CD11b+/CD11c+/B220+), or classic DCs (cDCs, I-A/I-E+/F4/80-/CD11b+/CD11c+/B220-) were strikingly decreased in the CNS of MOG-immunized mice with the administration of prophylactic but not semi-therapeutic dulaglutide treatment as compared to vehicle control, respectively (Figure 6B), insinuating that dulaglutide administration might influence the DCs populations in the CNS of EAE mice to enhance the encephalitogenicity of T cell subsets. Moreover, we also measured the percentage and cell number of total macrophages (I-A/I-E+/F4/80+/CD11b+), M1-like (I-A/I-E+/F4/80+/CD11b+/CD11c+), or M2-like (I-A/I-E+/F4/80+/CD11b+/CD11c-) macrophages in the CNS of EAE mice upon treatment of vehicle control, prophylactic, or semi-therapeutic dulaglutide, respectively. The percentages of macrophage subsets were similar among mice received different regimens (Figure 6C), respectively. We found that prophylactic dulaglutide treatment significantly reduced the cell numbers of total, M1-like macrophages, and M2-like macrophages in the CNS (Figure 6D). Nevertheless, semi-therapeutic dulaglutide treatment failed to affect the development of macrophages in CNS of EAE mice, as compared to vehicle control (Figure 6D). Taken together, these findings support that dulaglutide may have a modulatory effect in the regulation of DCs and macrophages in the CNS during the pathogenesis of autoimmune encephalomyelitis.

## 3. Discussion

In our study, we demonstrated that dulaglutide, a well-known GLP-1 RA, potently possesses protective effects on the pathogenesis of autoimmune encephalomyelitis via immune-modulation of CD4-positive T helper cell lineage subsets in the CNS parenchyma. Our investigations suggested that dulaglutide treatment suppresses the percentage of encephalitogenic Th1/Th17 cell subset and the GM-CSF production of Th1 cells in the CNS of EAE mice. The dulaglutide failed to affect the chemotactic capabilities of encephalitogenic Th1 and Th17 subsets into the CNS of EAE mice, whereas prophylactic dulaglutide treatment immensely diminishes the populations of DCs and macrophages in the CNS parenchyma of EAE mice. Conclusively, we demonstrated that dulaglutide administration rescues the clinical manifestations of EAE mice, in which the differentiation of encephalitogenic tissue-infiltrating Th1/Th17 subset and the pathogenicity of Th1 cell are dramatically alleviated in the CNS, in part, via the downregulation of APCs populations.

Despite its original purpose for blood glucose control, the role of GLP-1 RA on immune modulation has been increasingly recognized [28]. EAE, which mimic the human multiple sclerosis, is established as a CD4+ T cell-mediated disease model in the rodent. Despite its establishment as a neuroprotector, the role of GLP-1 RA in the pathogenic activity of T cells of EAE remains to be elucidated. Liraglutide is a GLP-1 RA, and its treatment delays the disease onset of EAE in myelin basic protein (MBP)-immunized Lewis Rats, where resistance against oxidative stress is improved in the brain of the drug-treated animal at the early stage of disease burden [7]. Liraglutide administration, indeed, enhanced the anti-oxidant manganese superoxide dismutase (MnSOD) and diminishes amyloid precursor protein (APP) expression in brain parenchyma of EAE rat at day 11 after MBP immunization. Another report revealed that treatment of Exendin-4, also a well-known GLP-1 RA, ameliorates the severity of EAE in MOG-immunized mice by the therapeutic administration from day 29 after MOG immunization [8]. Exendin-4 administration significantly attenuates the NF-κB signaling in the CNS and also represses the activation of microglia cells upon LPS stimulation. We administered GLP-1 RA dulaglutide to EAE mice by prophylactic and semi-therapeutic protocols. Consistent with previous results [7], prophylactic dulaglutide treatment dramatically delays the disease onset of EAE. Once prophylactic dulaglutide treatment was halted, we determined that the clinical manifestations of EAE start to occur likely at one week after the latest dulaglutide administration. In our semi-therapeutic dulaglutide treatment, the severity of EAE is remarkably attenuated, consistent with the report [8] that treatment of Exendin-4 strikingly alleviates the severity of EAE burden at late effector phase. However, semi-therapeutic dulaglutide treatment scarcely maintains its original protective effect on the late course of EAE disease. In summary, GLP-1 RA treatment, indeed, ameliorates the clinical outcomes of EAE, providing an essential insight into potential future therapy against human MS.

Our histological analysis revealed that the extent of CNS-infiltrating mononuclear cells, vacuolar degeneration, and demyelination of spinal cord are alleviated in dulaglutide treated-EAE mice, as shown by deep blue staining with Luxol Fast Blue at the lateral, dorsal and ventral funiculi. Demyelination of motor neurons in these funiculi leads to ascending paralysis of the limbs and the tail in EAE mice [29]. These results are highly correlated with the clinical manifestations and severity of EAE at day 14 after MOG immunization. Isolating mononuclear cells from the pooled brain and spinal cord, we found that the development of highly encephalitogenic Th1/Th17 cells in the CNS are significantly suppressed in prophylactic and semi-therapeutic dulaglutide-treated mice, despite lower absolute numbers of CNS-infiltrating mononuclear cells were present in CNS. These values are consistent with lower clinical scores in EAE mice receiving either prophylactic or semi-therapeutic dulaglutide administration. Briefly, Th1/Th17 cell is a T cell subset co-expressing IFN-γ and IL-17A that is present in CNS of EAE mice [14]. Th1/Th17 subsets, driven by IL-12 or IL-23 induction [14,30], possess higher encephalitogenicity than either Th1 or Th17 cells. Our study also indicated that GM-CSF secretion of encephalitogenic Th1 cell is diminished in prophylactic dulaglutide treatment, suggesting a critical role in the modulation of Th1 pathogenicity in the CNS. GM-CSF production of encephalitogenic Th1 cells is independent of IL-23 and dependent on IL-12 [17,31]. However, GM-CSF expression of encephalitogenic Th17 cells is driven by the effector function of IL-23 [16,32]. Our results revealed that GM-CSF secretion of Th17 cell is barely different under the treatment of either prophylactic or semi-therapeutic manner. Taken together, we aim to decipher whether dulaglutide treatment affects the production of pro-inflammatory cytokines, such as IL-12 or IL-23, from APCs in the CNS parenchyma, and thus consequently leads to the effect of dulaglutide on the differentiation of Th1/Th17 cells and pathogenicity of Th1 cells. We proposed a possible hypothesis that a profound decrease of APC numbers in the CNS parenchyma of dulaglutide-treated EAE mice represents lower expression levels of pro-inflammatory cytokines in the CNS of the dulaglutide-administrated animal, possibly dampening the development but not the quantities of cytokine production of T helper cells. Moreover, another GLP-1 RA Exendin-4 treatment causes the inactivation of NF-κB signaling in the spinal cord and downregulates levels of IL-17, IL-1β, IL-6, and TNF-α mRNA [8]. Indisputably, the signaling axis of GLP-1 and its receptor GLP-1R may be crucial in the regulation of cytokine expression from APCs or T cells.

GLP-1 receptor signaling is implicated in the regulation of lymphocyte proliferation and the maintenance of peripheral regulatory T cells. Glp1 knockout mice exhibited hyper-proliferative lymphocytes in response to mitogenic stimuli and a lower percentage of peripheral regulatory T cells [24]. In our study, we have analyzed the percentage of Treg subset (CD45^hi^/CD3^+^/CD4^+^/FOXP3^+^) in CNS. The results show no significant differences between vehicle, prophylactic treatment, and semi-therapeutic treatment. Whether GLP-1 RA affected the cytokine-producing ability of the infiltrating Treg in the CNS needs to be further clarified. Prophylactic treatment significantly reduces the infiltration of APCs, including dendritic cells (I-A/I-E+/F4/80-/CD11b+CD11c+) and macrophages (I-A/I-E+/F4/80+/CD11b+) in the CNS, which may affect the development of autoreactive T cells. Similar with our results, treatment of GLP-1 RA [33] or DPP4 inhibitors [34] reduces adipose macrophage infiltration in an obese mice model. Treatment of monocyte-derived macrophage (HMDM) with GLP-1 RA, Exenatide, induces activation of signal transducer and activator of transcription 3 (STAT3) signaling and M2 polarization through GLP-1 receptor [35]. Nevertheless, our findings indicate that M2-like macrophage is scarcely affected in the CNS of dulaglutide-administrated EAE mice as compared to vehicle control-treated mice. Moreover, treatment of cultured macrophage THP-1 cells with dipeptidyl peptidase-4 (DPP-4), an enzyme that degrades native GLP-1, suppresses NLRP3, TLR4, and IL-1β through inhibition of PKC [36]. These results indicated an anti-inflammatory role of GLP-1 through regulation of macrophages. Here we have shown that M1-like macrophage (I-A/I-E+/F4/80+/CD11b+/CD11c+) is significantly reduced in the CNS in the prophylactic treatment of dulaglutide; however, the M2-like macrophage (I-A/I-E+/F4/80+/CD11b+/CD11c-) is barely altered. The role of GLP-1 in the regulation of dendritic cells is found in studies using DPP4 inhibitors. Treatment of DPP4 inhibitor (gliptin) decreases dendritic cell maturation upon LPS-induced endotoxemia [37]. Consistent with previous data, our findings revealed that prophylactic dulaglutide treatment significantly reduces the populations of dendritic cells, further suggesting lower functions of DCs to reactivate the autoreactive T cells in CNS.

In this study, EAE has been induced in C57BL/6J stain by MOG_35–55_, which is the most widely used model of EAE. The disease course of this model mainly follows a relapse-remitting or chronic disease course during the 40 days of observation [38]. Notably, establishing of EAE on the non-obese diabetic (NOD) background would lead to the development of relapse-remitting to a chronic progressive stage from 20 to 70 days after MOG immunization [39], which is considered closer to the clinical disease course of MS patients [40]. Non-obese diabetic (NOD) mice strain is an autoreactive CD8+ T cell-driven disease model, which developed diabetes spontaneously due to the destruction of the pancreatic insulin-producing beta cells. In addition, accumulating evidence suggest that CD8+ T cell also plays a key role in MS pathology [40]. Thus, it will provide more mechanistic insight on T cell regulation and understanding of GLP-1 mimetics on the interaction of MS and diabetes by using NOD for EAE induction.

In conclusion, here we have provided evidence that the protective effect of GLP-1 RA on EAE, in addition to the neuroprotection effect as described in previous studies, is also contributed by immune regulation during the pathogenic stage. Administrating of GLP-1 RA early at disease induction stage dramatically attenuates the activation of infiltrating/resident immune cells. Semi-therapeutic treatment of GLP-1 RA has a relatively moderate but similar trend on immune regulation compared with early treatment. Whether the non-significance of the semi-therapeutic group is due to the limited sample size warrant further study. In addition to the regulation of macrophages and DCs, this is the first report that GLP-1 RA ameliorates EAE through affecting the development of Th1/Th17 and Th1 pathogenicity. These results provide more mechanistic insight for the application of GLP-1 RA on MS treatment.

## 4. Materials and Methods

### 4.1. Animals and Experimental Design

Animal Use Protocol (IACUC-105014) was approved by the Kaohsiung Medical University-Institutional Animal Care and Use Committee at 22 March 2016. Male mice (aged 8~10 weeks) were purchased from National Laboratory Animal Center (Taiwan). The mice were housed in an Association for Assessment and Accreditation of Laboratory Animal Care International (AAALAC)-accredited facility of Kaohsiung Medical University, which the room temperature was (21 + 2) °C, the humidity was 55~70% in a 12:12 h dark/light cycle After 2 weeks of adaptation, the mice were immunized with MOG and randomly divided into 3 groups: vehicle (*n* = 3), semi-therapeutic group (*n* = 3), prophylactic treatment (*n* = 4). Three batches of independent experiments were performed and the results were collected for statistical analysis. Dulaglutide (Eli Lilly and Company, Indianapolis, IN, USA) were administrated at the dosage of 0.18 mg/kg by subcutaneous (*s.c.*) injection. For prophylactic treatment, mice were administrated with dulaglutide after MOG immunization twice per week from day 0 for a total of two weeks. For semi-therapeutic treatment, dulaglutide were administered twice per week from day 9 after MOG immunization for a total of three weeks. For determining the effect of dulaglutide on EAE pathogenic process, the mice were followed up for 4 weeks from MOG-immunization to determine the EAE disease incidence, day of onset, maximal clinical scores, and duration with the maximal clinical score. For immune cell subset analysis, three groups of mice were all sacrificed at day 14 from MOG-immunization, and the brain and spinal cord were harvested for further analysis.

### 4.2. EAE Induction and Evaluation of Clinical Outcomes

10 to 12-week-old C57BL/6J mice were immunized subcutaneously on day 0 with 100 μg/per mouse of MOG_35–55_ peptide emulsified in complete Freund’s adjuvant supplemented with 400 mg/mL Mycobacterium tuberculosis H37Ra (Difco, Kansas City, MO, USA) [41]. Each mouse was subsequently administered 250 ng pertussis toxin (List Biological Laboratories, Campbell, CA, USA) by intraperitoneal (*i.p.*) injection on days 0 and 2 after MOG_35–55_ peptide immunization. The EAE clinical manifestations were evaluated by daily assignment of scores from 0 to 5 as follows: 0, no clinical sign; 0.5, partial weakness of limb tail; 1, complete paralysis of tail; 1.5, paralysis of tail and waddling gait; 2, paralysis of one hind limb; 2.5, paralysis of one hind limb and partial paralysis of the other hind limb; 3, paralysis of both hind limbs; 3.5, forelimb weakness; 4, forelimb paralysis; 5, moribund or death.

### 4.3. Tissue Preparation

The mice were sacrificed by CO_2_ asphyxiation at day 14 of MOG-immunization for flow cytometry and histological analysis. For flow cytometry analysis, parenchymal mononuclear cells were isolated from pooled brain and spinal cord after the transcardiac perfusion of PBS buffer as described previously [42,43]. Tissues of CNS were mechanically dissociated through the mesh strainer, and homogenized cells were subsequently fractionated on a 70%–30% Percoll gradient by centrifugation at 500× g for 30 min at room temperature with the lowest acceleration, without deceleration. The parenchymal infiltrating mononuclear cells were collected from the interface phase and then washed twice with 1x HBSS (Hank’s Balanced Salt Solution) buffer without phenol red. Finally, cells were counted and then stimulated with 1x Cell Stimulation Cocktail (plus protein transport inhibitors) (eBiosciences, San Diego City, CA, USA) for 4 h at 37 °C in a humidified 5% CO_2_ atmosphere of the incubator. The cocktail-stimulated mononuclear cells were treated by the procedures of intracellular staining and flow cytometry as mentioned above. For histological analysis, the cervical spinal cord was removed after transcardiac PBS perfusion, and immediately fixed by 4% paraformaldehyde and embedded in paraffin for tissue slice preparation.

### 4.4. Flow Cytometry

Surface makers of cells were stained with fluorochrome-conjugated antibodies for murine CD3ε (145-2C11), CD4 (RM4-5), CD45 (30F11) (purchased from eBiosciences, San Diego City, CA, USA), CD11b (M1/70), CD11c (N418), CXCR3 (CXCR3-173), I-A/I-E (M5/114.15.2), F4/80 (BM8), B220 (CD45R) (RA3-6B2), or CCR6 (29-2L17) (purchased from Biolegend, San Diego City, CA, USA) (Table 2) on ice for 30 min. Cells were washed once and then were suspended in FACS (Fluorescence-Activated Cell Sorter) running buffer. The flow cytometry analysis of cells was performed with the BD LSRII (BD Biosciences, San Jose City, CA, USA). Finally, Flow Jo software was used for data analysis.

### 4.5. Intracellular Cytokine Staining

Cells were stimulated with 1x Cell Stimulation Cocktail (plus protein transport inhibitors) (eBiosciences, San Diego, CA, USA), containing phorbol 12-myristate 13-acetate (PMA), ionomycin, brefeldin A and monensin, for 4–6 h at 37 °C in a humidified 5% CO_2_ atmosphere of the incubator. Surface makers of cells were stained with fluorochrome-conjugated antibodies specific for murine CD3ε (145-2C11) CD4 (RM4-5) (purchased from eBiosciences, San Diego, CA, USA), or CD45 (30F11) (purchased from eBiosciences, San Diego, CA, USA) on ice for 30 min. Cells were washed once with 1x FACS buffer and then fixed with IC fixation buffer (eBiosciences, San Diego, CA, USA) for 20 min at room temperature. Subsequently, the already fixed cells were washed with 1x permeabilization buffer once and then those cells were stained with fluorochrome-conjugated antibodies for murine IFN-γ (XMG1.2), TNF-α (MP6-XT22), (purchased from Biolegend, San Diego, CA, USA), FoxP3 (FJK-16s), IL-10 (JES5-16E3), IL-17A (TC11-18H10.1), and GM-CSF (MP1-33E9) (purchased from eBiosciences, San Diego, CA, USA) (Table 2) for one hour at room temperature. Washing cells with 1x permeabilization buffer once and then suspending them in FACS buffer, the flow cytometry analysis of cells was performed with BD LSRII (BD Biosciences, San Jose, CA, USA). Ultimately, Flow Jo software was employed for data analysis. For the purpose of staining cells, we designed four sets of antibodies in our experiments: 1. CD45/CD3ε/CD4/FoxP3; 2. CD45/CD3ε/I-A/I-E/F4/80/CD11b/CD11c/B220; 3. CD45/CD3ε/CD4/IFN-γ/IL-17A/GM-CSF/TNF-α/IL-10; 4. CD45/CD3ε/CD4/IFN-γ/IL-17A/CXCR3/CCR6.

### 4.6. Histological Analysis of Spinal Cord

The cervical spinal cord was cross-sectioned at 5 μm thickness and stained with Hematoxylin and Eosin (H&E) and Luxol Fast Blue (LFB) for identification of parenchymal infiltrating mononuclear cells and myelin integrity [29], respectively. The tissue sections were examined under a light microscope to evaluate demyelination and inflammatory cell infiltration in parenchymal cells of the spinal cord.

### 4.7. Statistical Analysis

The non-parametric Kruskal–Wallis test followed by post-hoc test, Dunn’s multiple comparisons analysis, were analyzed for statistical analysis of all experiments in this study.

## Figures and Tables

**Table 1:** GLP-1 RA dulaglutide administration extremely influences the disease progression of EAE.

Treatment	EAE Incidence	Day of Onset (Mean ± SEM)	Maximal Clinical Score (Mean ± SEM)	Duration with Maximal Clinical Score (Mean ± SEM)
Vehicle	8/8	10.88 ± 0.5154	3.781 ± 0.2083	2.375 ± 0.3239
Prophylactic	11/12	18.82 ± 1.256 ****	2.021 ± 0.3053 ***	2.7 ± 0.5175
Semi-therapeutic	9/9	14.0 ± 0.7601 *	2.889 ± 0.2170 *	2.0 ± 0.2887

**** *p* < 0.0001; *** *p* < 0.001; * *p* < 0.05 by non-parametric Kruskal-Wallis test followed by post-hoc analysis.

**Table 2:** Antibodies used in the experiments of surface and intracellular FACS staining.

Antigen	Fluorochrome	Clone	Manufacturer
CD45	PE-Cy7	30-F11	eBioscience
CD3ε	APC-eFluor 780	145-2C11	eBioscience
CD4	Alexa Fluor 700	RM4-5	eBioscience
FoxP3	PE	FJK-16s	eBioscience
IL-17A	PerCP-Cy5.5	eBio17B7	eBioscience
GM-CSF	PE	MP1-22E9	eBioscience
CXCR3	FITC	CXCR3-173	eBioscience
I-A/I-E	APC	M5/114.15.2	eBioscience
IFN-γ	FITC or BV421	XMG1.2	Biolegend
IL-10	PE-Dazzle 594	JES5-16E3	Biolegend
TNF-α	APC	MP6-XT22	Biolegend
CCR6	APC	29-2L17	Biolegend
F4/80	BV421	BM8	Biolegend
CD11b	PE	M1/70	Biolegend
CD11c	FITC	N418	Biolegend
B220 (CD45R)	PerCP-Cy5.5	RA3-6B2	Biolegend

## Abbreviations

GLP-1	Glucagon-like peptide-1
GLP-1 RA	GLP-1 receptor agonist
EAE	Experimental autoimmune encephalomyelitis
MS	Multiple sclerosis
CNS	Central nervous system
Th1	Type 1 helper T
Th17	Type 17 helper T
Th1/Th17	IFN-γ/IL-17A-doubly-secreting CD4 T
MFI	Mean fluorescence index
GM-CSF	Granulocyte-macrophage-colony-stimulating factor
TNFα	Tumor necrosis factor α
IL	Interleukin
Tr1	T regulatory type 1
APCs	Antigen-presenting cells
DCs	Dendritic cells
pDCs	Plasmacytoid DCs
cDCs	Classic DCs
MnSOD	Manganese superoxide dismutase
APP	Amyloid precursor protein
MOG	Myelin oligodendrocyte glycoprotein
CFA	Complete Freund’s adjuvant
MBP	Myelin basic protein
H&E	Hematoxylin and Eosin
LFB	Luxol Fast Blue
HMDM	Human monocyte-derived macrophage
STAT3	Signal transducer and activator of transcription 3
DPP-4	Dipeptidyl peptidase-4
AAALAC	Association for Assessment and Accreditation of Laboratory Animal Care
s.c.	Aubcutaneous
i.p.	Intraperitoneal
PMA	Phorbol 12-myristate 13-acetate
